# Supplementary material for: The causal relationship between the human gut microbiota and pyogenic arthritis: a Mendelian randomization study
Source: Front Cell Infect Microbiol. 2024 Nov 26;14:1452480. doi: 10.3389/fcimb.2024.1452480 (PMC11629706; doi:10.3389/fcimb.2024.1452480)
Supplement: Supplementary file 1 [file DataSheet1.zip › mendelian test/ebi-a-GCST90027851.csv_forest.pdf]

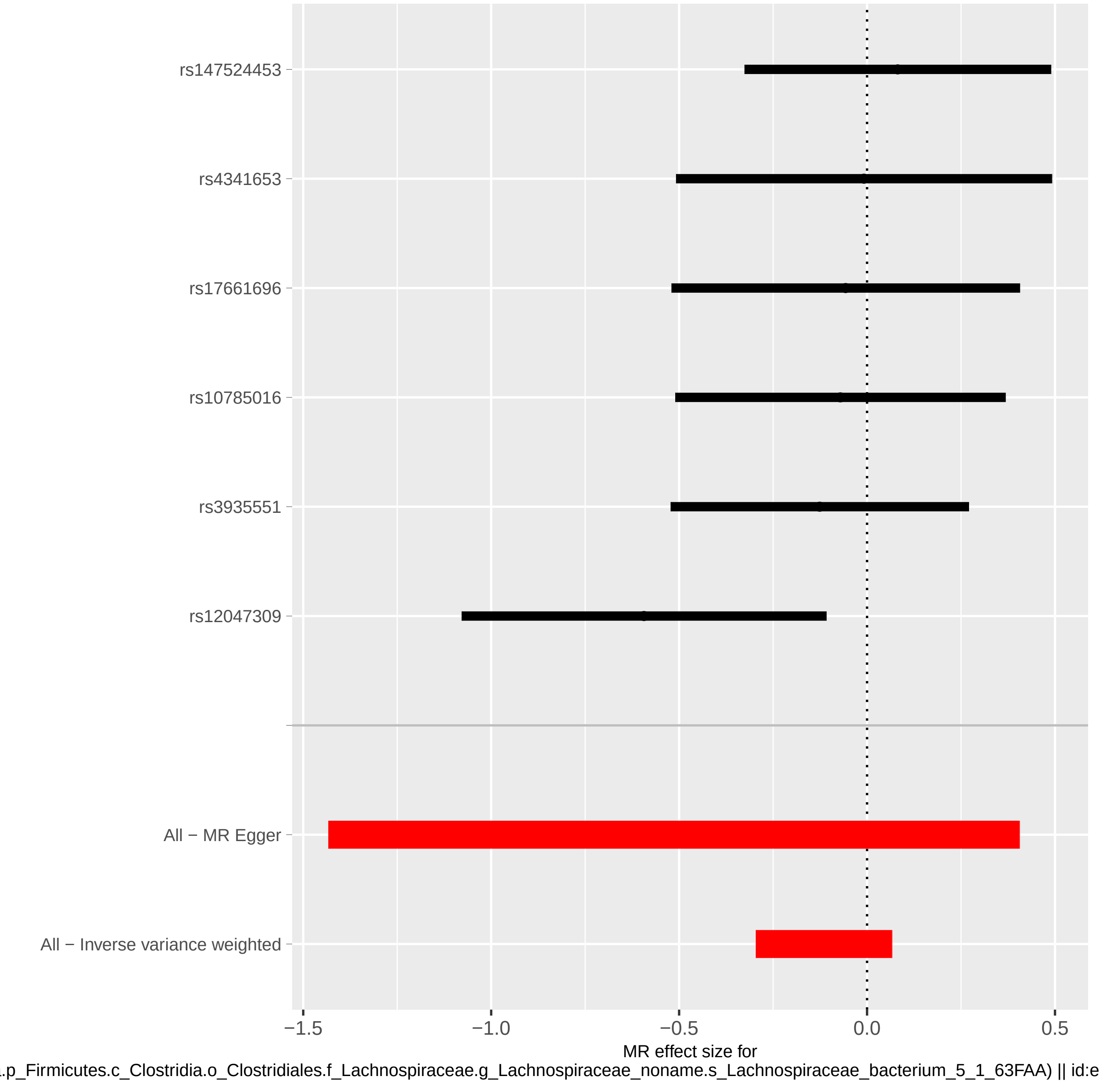
rs147524453
rs4341653
rs17661696
rs10785016
rs3935551
rs12047309
All − MR Egger
All − Inverse variance weighted
−1.5
−1.0
−0.5
0.0
0.5
MR effect size for
.p_Firmicutes.c_Clostridia.o_Clostridiales.f_Lachnospiraceae.g_Lachnospiraceae_noname.s_Lachnospiraceae_bacterium_5_1_63FAA) || id:e
